# Uptake and transport of antibiotic kasugamycin in castor bean (*Ricinus communis* L.) seedlings

**DOI:** 10.3389/fmicb.2022.948171

**Published:** 2022-08-10

**Authors:** Hongzhen Zhang, Chenghua Zhang, Xiaolong Xiang, Qilun Zhang, Wei Zhao, Guoyu Wei, Anlong Hu

**Affiliations:** ^1^College of Agriculture, Guizhou University, Guiyang, Guizhou, China; ^2^Shandong Academy of Agricultural Sciences, Jinan, Shandong, China; ^3^Forestry Bureau of Wuchuan County, Zunyi, Guizhou, China

**Keywords:** kasugamycin, antibiotic, uptake, plant, transport

## Abstract

Kasugamycin (KSM), an aminoglycoside antibiotic, has been widely used for the management of plant diseases, especially for the control of rice blast in Asia. However, its uptake mechanism and transport in plants are still obscure. The castor bean (*Ricinus communis* L.) seeding, a model plant for phloem transport, was used to study the mechanism of uptake and transport of KSM. Results showed that cotyledon-applied KSM could transport into the phloem and distributed in root and shoot of plant. The temperature, concentration, and pH had significant effects on the uptake of KSM, indicating that the uptake of KSM was mediated by an active carrier system. Compared with the control, competitive inhibitors of sugar transporters D-glucose, D-chiro-inositol, and phloridzin inhibited 71.03%, 67.95%, and 61.73% uptake of KSM, respectively. Energy inhibitor dinitrophenol (DNP) and carbonyl cyanide chlorophenylhydrazone (CCCP) also affected the uptake of KSM, and the inhibition rates were 34.23% and 48.06%. All the results showed that the uptake of KSM was mediated by a sugar transporter, and it could transport from shoot to root in plants *via* the phloem. The study preliminary elucidated the plant–microbe interactions in the context of the transport of microbial secondary metabolites in plants. It has certain significance for scientific application of antibiotics and biological control of plant diseases and provides theoretical basis for the development of bidirectional transport pesticides.

## Introduction

Kasugamycin (KSM), an aminoglycoside antibiotic isolated from Streptomyces kasugaensis (Umezawa et al., 1965), exhibits inhibitory activity on protein biosynthesis with low toxicity to humans, animals, and plants (Duffin and Seifert, 2009). It is widely used in the control of many plant diseases, such as leaf spot, fire blight, and bacterial diseases in various crops, especially in the control of rice blast caused by *Piricularia oryzae* (Li et al., 2020a; Amoghavarsha et al., 2021).

Ishiyama et al. (1967) have shown that the translocation of KSM in plants is bidirectional. Ishiyama applied 14C-marked KSM on the abaxial surface of rice leaves, and autoradiography showed that the KSM could penetrate and transfer into foliar tissues; eventually, it was distributed in the shoots and roots of rice. Applied to the middle leaf of tobacco, KSM was detected in the upper leaves, lower leaves, and the roots of tobacco (He, 2010). Because the long-distance transport of xenobiotics from shoots to roots in plants must take place in the phloem, these studies suggest that KSM has phloem mobility properties. The above studies only showed that KSM has the property bidirectional translocation in plants, but there is a lack of in-depth research on its uptake mechanism and transport path.

Phloem mobile pesticides can move toward roots and protect tissues that don’t expose to pesticides, such as root or vascular. This mobility can avoid the influence of soil factors on pesticides and allow for reduced environmental pollution and improvements in the management of soil-borne diseases compared with the pesticide application method of root irrigation. To date, the number of pesticides that have demonstrated phloem mobility in plants is very limited, exemplified by current pesticides such as fosetyl-Al (Leconte et al., 1988), metalaxy (Leconte et al., 1988), glyphosate (Gougler and Geiger, 1981), 2,4-dichlorophenoxyacetic acid (Crafts, 1956), and spirotetramat (Nauen et al., 2008).

Many efforts have been made in our understanding of the phloem mobility of pesticides. Phloem mobility of plant can be significantly affected by the physico chemical properties of pesticides and plant parameters (Grayson and Kleier, 1990; Kleier, 1994; Kleier and HSu, 1996). However, this representation cannot explain the phloem mobility of some pesticides that do not conform to it, such as glyphosate and paraquat. Because uptake of most phloem mobile pesticides is carrier mediated. The uptake of the herbicide glyphosate by broad bean was investigated using protoplasts of broad bean leaves, and it was concluded that glyphosate could be absorbed *via* a phosphate transporter of the plasma membrane (Denis and Delrot, 1993). Phosphate transporters mediate the uptake of phosphite, an active metabolite of fosetyl-Al in plants (Achary et al., 2017). There are two mechanisms for the uptake of 2, 4-dichlorophenoxyacetic acid in maize root protoplasts: an ion-trap mechanism and active transport mediated by an auxin carrier (Kasai and Bayer, 1995). Paraquat is translocated by a diamine carrier (Hart et al., 1992). An amino acid transporter-like protein plays an essential role in thiamethoxam uptake and its systemic distribution in rice (Xiao et al., 2022). The plasma membrane is a barrier for xenobiotics to enter the symplast, but carriers on the plasma membrane can promote uptake, as the above studies show.

4-Chloro-7-nitro-1,2,3-benzoxadiazole (NBD-Cl) is usually used as a fluorescent probe to trace the uptake and translocation of compounds in organisms (Li et al., 2020b; Jiang et al., 2021). Yoshioka et al. (1996) synthesized a fluorescent glucose conjugate 2-NBDG, which has the same uptake and transport mechanism as glucose, so it is widely used in the study of uptake and transport in animal and plant cells. Fluorescent conjugated KSM-NBD was synthesized by our team, and it could be traced in tissue of maize and tobacco under a fluorescence microscope (Zhang et al., 2021).

Energy-requiring and against concentration gradient uptake of aminoglycosides by bacteria indicates a carrier-mediated transport system (Taber et al., 1987). We studied the uptake of KSM by tobacco leaf discs, and the results were consistent with those of bacteria (Hu et al., 2012). These considerations led us to investigate the possibility that KSM uptake into plant cells may in part be mediated *via* a carrier. Castor bean (*Ricinus communis* L.) is a classic model plant for phloem transport (Schmidke and Stephan, 1995; Schmidke et al., 1999; Melicherová et al., 2020). Phloem exudate collected from castor bean cotyledons after cutting the hypocotyl was used to analyze the uptake and transport of KSM.

Soil-borne pathogenic organisms can have severe detrimental effects on crop growth and yield production and represent a serious threat to food security (Chen et al., 2020; Witzel and Berger, 2020). When applied directly to soil to control root diseases, most of the pesticides will be absorbed by the soil, resulting in low pesticide activity and environmental pollution. Developing pesticides that can be transported from the upper part of the plant to the roots is an innovative way to solve the problem. The translocation of KSM to the roots of plants gives us some insights. Explore of the uptake mechanism and transport pathway of KSM in plant will lay theoretical basis for research on the development of phloem mobile and root-target pesticides.

## Materials and methods

### Chemicals

KSM (90%), phloridzin, dinitrophenol (DNP), carbonyl cyanide chlorophenylhydrazone (CCCP), D-glucose, and D-chiro-inositol (DCI) were all purchased from Aladdin Reagent Co. Ltd. (Shanghai, China).

### Plant materials

Castor bean seeds (Castor bean No. 9, obtained from the Agricultural Science Academy of Zibo Shandong, China) were kept in humid cotton at 27°C for 24 h and then transferred to an artificial incubator for cultivation. Seedlings were grown at 80% relative humidity and 27°C ± 1°C in light (14 h photoperiod) (Chollet et al., 2004). Seven and 14 day old average-sized seedlings were selected for further experiments.

### Long-distance transport and distribution

**Figure 1:**
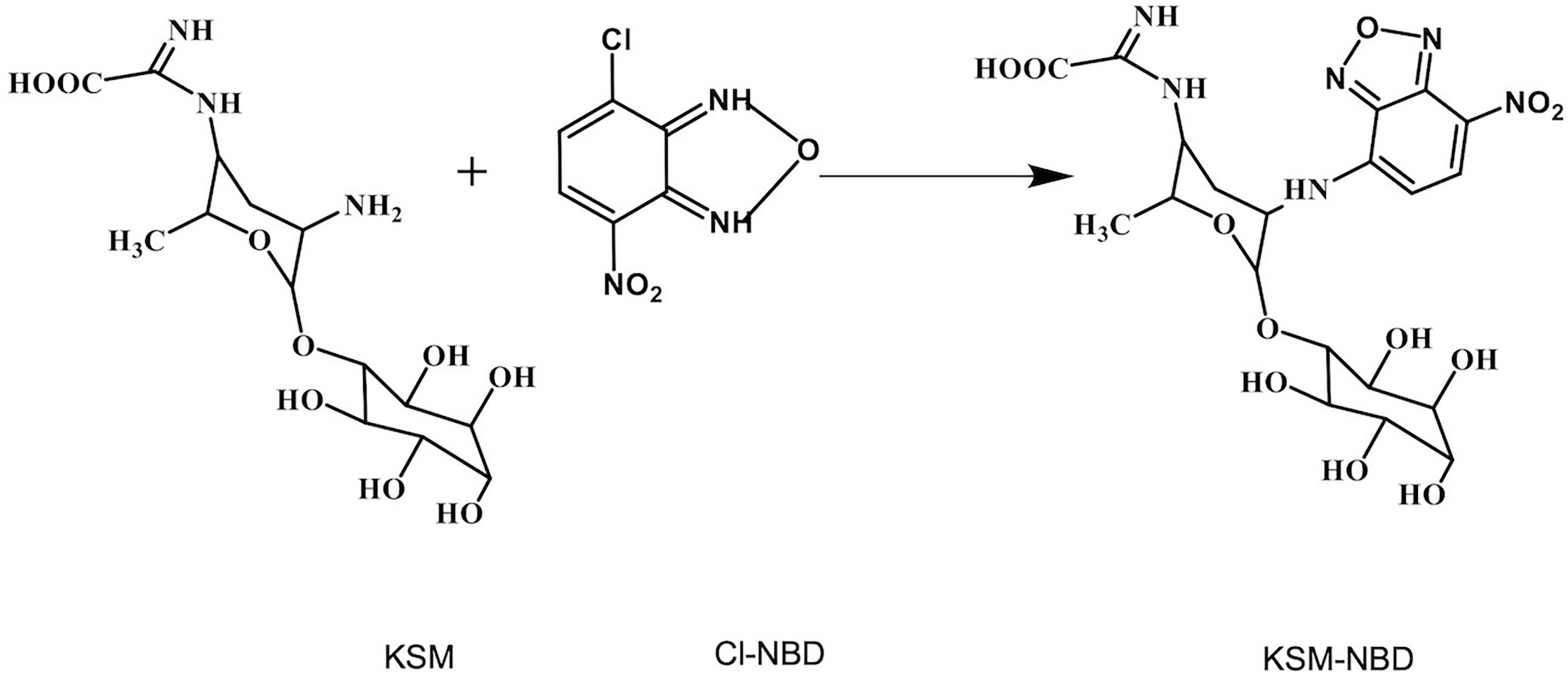
Schematic diagram of KSM-NBD synthesis by KSM and NBD-Cl.

**Figure 2:**
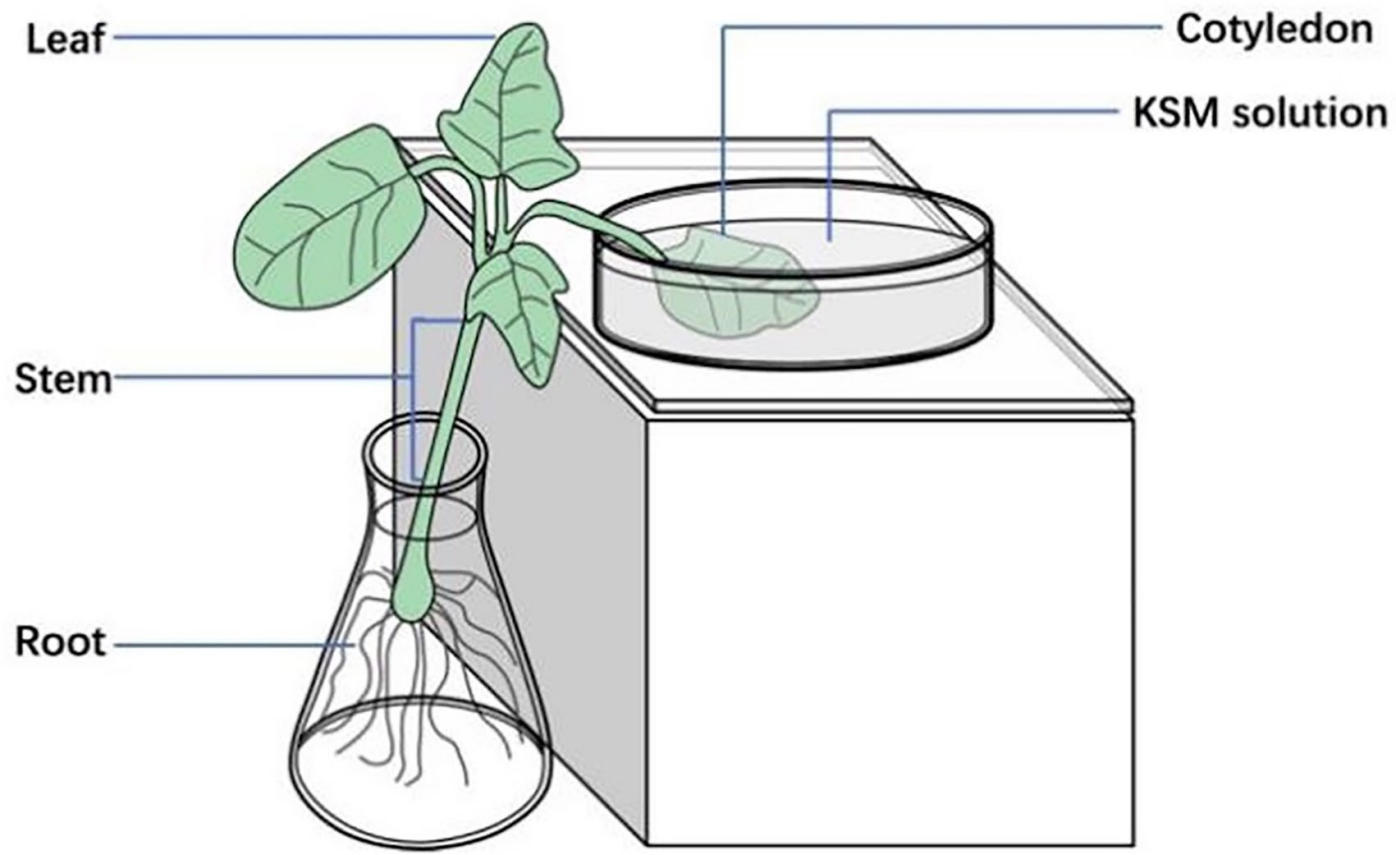
Diagrammatic representation of uptake of KSM by 2-week-old castor beans. Castor beans grown in an artificial incubator for 2 weeks were cultivated in Hoagland solution in a conical flask. One cotyledon was dipped into a solution containing 0.1 mM KSM.

Fluorescent conjugate KSM-NBD was synthesized by our team (Zhang et al., 2021; Figure 1). Castor beans grown in an artificial incubator for 2 weeks were cultivated in a hydroponic system with Hoagland solution in a conical flask. The cotyledon was incubated in a solution containing 0.1 mM KSM and KSM-NBD, respectively (pH = 5.5, 90% ± 5% RH, 27°C, 14 h photoperiod; Figure 2). The top of the leaf, stems, and roots were collected after 24 and 48 h, respectively. Samples (1 g) were ground in a mortar with 4 ml solution (methanol:water = 9:1 v/v) and then transferred to a centrifuge tube. After centrifugation in a refrigerated centrifuge at 1,500 RPM for 15 min at 5°C (ST16 R, Thermo Fisher Scientific, Braunschweig, Germany), the supernatant was stored in a sample vial after filtration through a 0.22 μm nylon filter for HPLC–MS/MS analysis. Samples which cultivated in KSM-NBD are used for immunofluorescence observation. Observe the slices under a fluorescence microscope (NIKON ECLIPSE C1, Nikon, Tokyo, Japan) and collect images. The experiment was repeated three times with one seedling per replicate.

### Collection of phloem exudate

**Figure 3:**
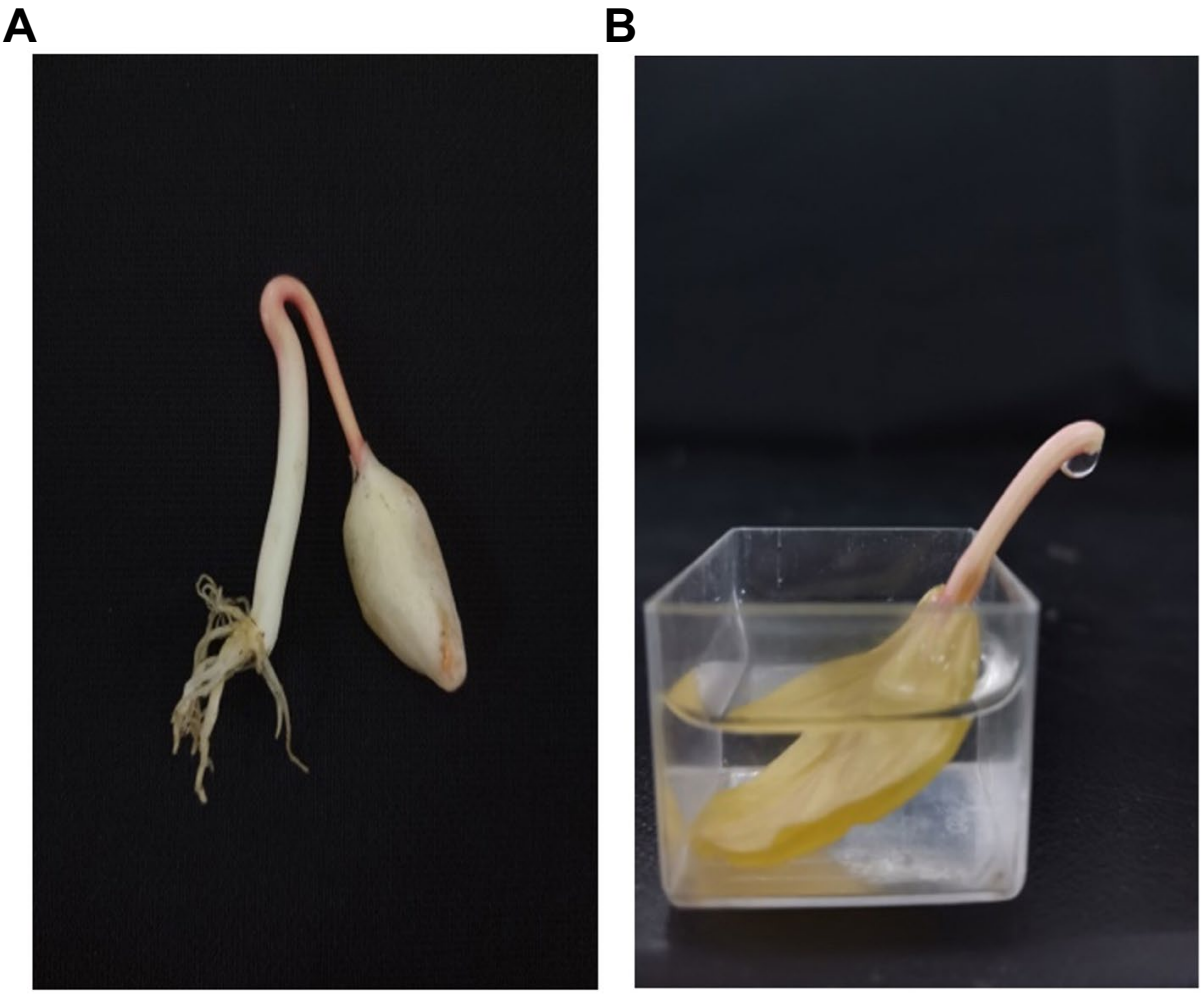
Collection of phloem exudate from castor bean seedlings. Seven-day-old seedlings with their cotyledons enclosed within the endosperm were used for phloem exudate collection **(A)**. The endosperm of the seedlings was carefully removed, and the cotyledon was incubated in a buffered solution. After 1 h of incubation, the hypocotyl was cut with a razor blade in the hook region **(B)**. Subsequently, the exudate was collected with a graduated capillary glass tube.

The phloem exudate was collected according to previously described methods with slight modifications (Lei et al., 2014). Seven days after sowing, average-sized seedlings with their cotyledons enclosed within the endosperm were used for phloem exudate collection. The endosperm of the seedlings was carefully removed, and the cotyledon was incubated in a buffered solution containing 20 mM 2-(4-morpholino, MES), 0.25 mM MgCl2, 0.5 mM CaCl2, without (control) or with other substances (according to the different experiments described below). After 1 h of incubation, the hypocotyl was cut with a razor blade in the hook region. Subsequently, the exudate was collected with a graduated capillary glass tube (Figure 3), and the collected exudate was stored at −20°C. The phloem exudate was analyzed after dilution with pure water (phloem exudate: pure water =1:4, v/v). All phloem exudate collection treatments were repeated 3 times with 8 seedlings per replicate to obtain sufficient exudate.

### Time course of uptake

The cotyledon which was grown in an artificial incubator for 1 week was incubated in a buffered solution (pH 5.5) with 0.1 mM KSM, and the phloem exudate was collected at 10, 20, 30, 50, 70, 90, 110, 130, and 150 min. The experiment was carried out in a growth chamber (25°C ± 2°C, RH 90% ± 5%).

### Concentration dependence of uptake

The cotyledon which was grown in an artificial incubator for 1 week was incubated in a buffered solution with 2, 1, 0.5, 0.25, 0.125, 0.0625, and 0.03125 mM KSM, and the phloem exudate of castor bean was collected after 1 h of incubation. All phloem exudates were collected for 1 h to calculate the speed of KSM exudate from the phloem of castor bean.

### Effect of temperature on uptake

We set temperatures of 14°C and 27°C and collected the phloem exudate at 60, 90, and 120 min after treatment. The cotyledon of castor bean which was grown in an artificial incubator for 1 week was incubated in buffer solution (pH 5.5) as described above in 2.2 with 0.1 mM KSM. Phloem exudate collected after 1 h of incubation was collected and then analyzed by HPLC–MS/MS. The temperature coefficient (Q10, the factor by which the reaction rate increases when the temperature is raised by ten degrees) equation is:


$$Q_{10}={\left[\frac{K_{2}}{K_{1}}\right]}^{10/\left(T_{2}-T_{1}\right)}$$

T2, higher temperature; T1, lower temperature; K2, uptake at higher temperature; K1, uptake at lower temperature.

### Effect of pH on uptake

The pH of the buffer solution with 0.1 mM KSM was adjusted to 5, 5.5, 6, 7, 8, and 9 with sodium hydroxide and citric acid, and then, the phloem exudate of castor bean was collected as described in section 2.2 after 1 h of incubation.

### Effect of energy inhibitor and competitive substrate on uptake

The cotyledon which grown in an artificial incubator for 1 week was incubated in buffer solution and 0.1 mM KSM with energy inhibitors DNP (0.1 mM) and CCCP (0.05 mM), competitive substrate D-glucose (KSM: D-glucose = 1:40, 1:400, 1:4,000, molar ratio), D-chiro-inositol (KSM: D-chiro-inositol =1:3, 1:5, 1:7, molar ratio), and phlorizin (KSM: phloridzin = 1:5, 1:10, 1:20, molar ratio) for 1 h. The phloem exudate of castor bean was collected after an additional 1 h of incubation.

### Analysis of KSM

An Agilent 1,260 series HPLC equipped with a binary pump, autoplate sampler, column, and Agilent 6,470 Triple Quadrupole Mass Spectrometer (MS/MS; Agilent Technologies, United States) was used for the analysis of KSM. Chromatographic separations were achieved with a reversed-phase column (ZORBAX Eclipse, 150 mm × 4.6 mm, 5 μm) at 35°C. The mobile phase consisted of methanol and water (70:30, v/v) at a flow rate of 0.15 ml/min, and the injection volume was 5 μl. The mass spectrometer was operated in positive electrospray ionization mode (ESI+) using multiple reaction monitoring (MRM). The MS parameters of the target compound are as follows: spray voltage (IS+): 5,500 V; electrospray ion source temperature (TEM): 300°C; atomizing gas flow rate: 45 psi; collision cell inlet voltage (EP): 16 V; collision cell outlet voltage (CXP): 80 V; quantitative ion pair: m/z 380.3/200.3; and qualitative ion pair: m/z 380.3/112.1.

A series of standard solutions of KSM (2, 1, 0.25, 0.125, and 0.1 μg mL^−1^) were prepared in water to obtain a calibration curve. The linear equation of KSM was y = 542 × −24,276 (r^2^ = 0.998). Recovery studies were developed at three spiking levels of 0.1, 0.5, and 1 mg kg^−1^, and the individual mean recovery rates for KSM were 91%–98%. The lower limit of detection calculated as a signal/noise ratio = 3 for KSM was 0.001 μg L^−1^.

### Data analysis

Data Processing System v.7.05 (DPS, Zhejiang University, China) was used for data analysis, a one-way analysis of variance (ANOVA), followed by Duncan’s multiple range test was carried out to compare the differences among treatments (*p* < 0.01). The data were presented in the form of mean ± SE and Origin 2020 was used for plotting.

## Results

### Uptake and long-distance translocation of KSM in castor bean

**Figure 4:**
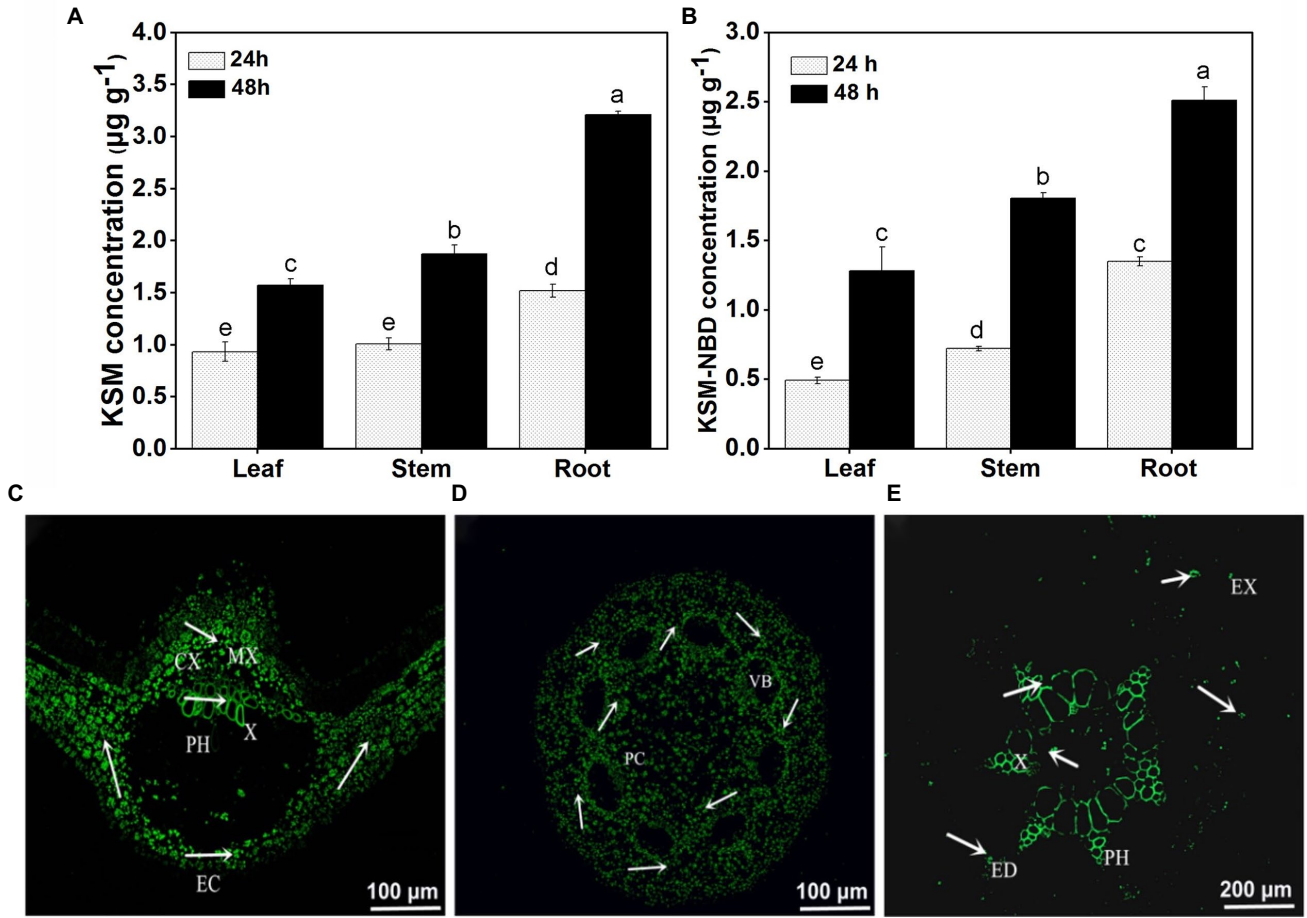
Content of KSM detected in 2-week-old castor bean 24 and 48 h post cotyledon dipped in 0.1 mM KSM **(A)** and KSM-NBD (**B**; mean ± SE, *n* = 2). Distribution of KSM-NBD in leaves **(C)**, stems **(D)**, and roots **(E)** of Castor bean. Green indicates the presence of fluorescent couplings; EC, epidermal cells; CX, cell wall space; MC, mesophyll cells; PH, phloem; X, Xylem. All images were obtained by fluorescence microscopy.

It was found that KSM and KSM-NBD could be detected in all parts of castor bean after cotyledon dipping in 0.1 mM KSM and KSM-NBD for 24 and 48 h (Figures 4A,B). The contents of KSM and KSM-NBD in the roots were higher than that in the leaves and stems, respectively. Fluorescence microscopic image of castor bean treated with KSM-NBD showed that KSM-NBD was mainly distributed in mesophyll cells and phloem of leaf (Figure 4C), protoplast cell of stem (Figure 4D), and phloem and xylem of root (Figure 4E). It indicated that the epidermal cells of cotyledons could uptake KSM-NBD from the culture medium. KSM-NBD could transport *via* the apoplast loading process (xylem) and can also reach the stems after being transported by the cotyledon process (phloem).

### Time course of KSM uptake by castor bean cotyledons

**Figure 5:**
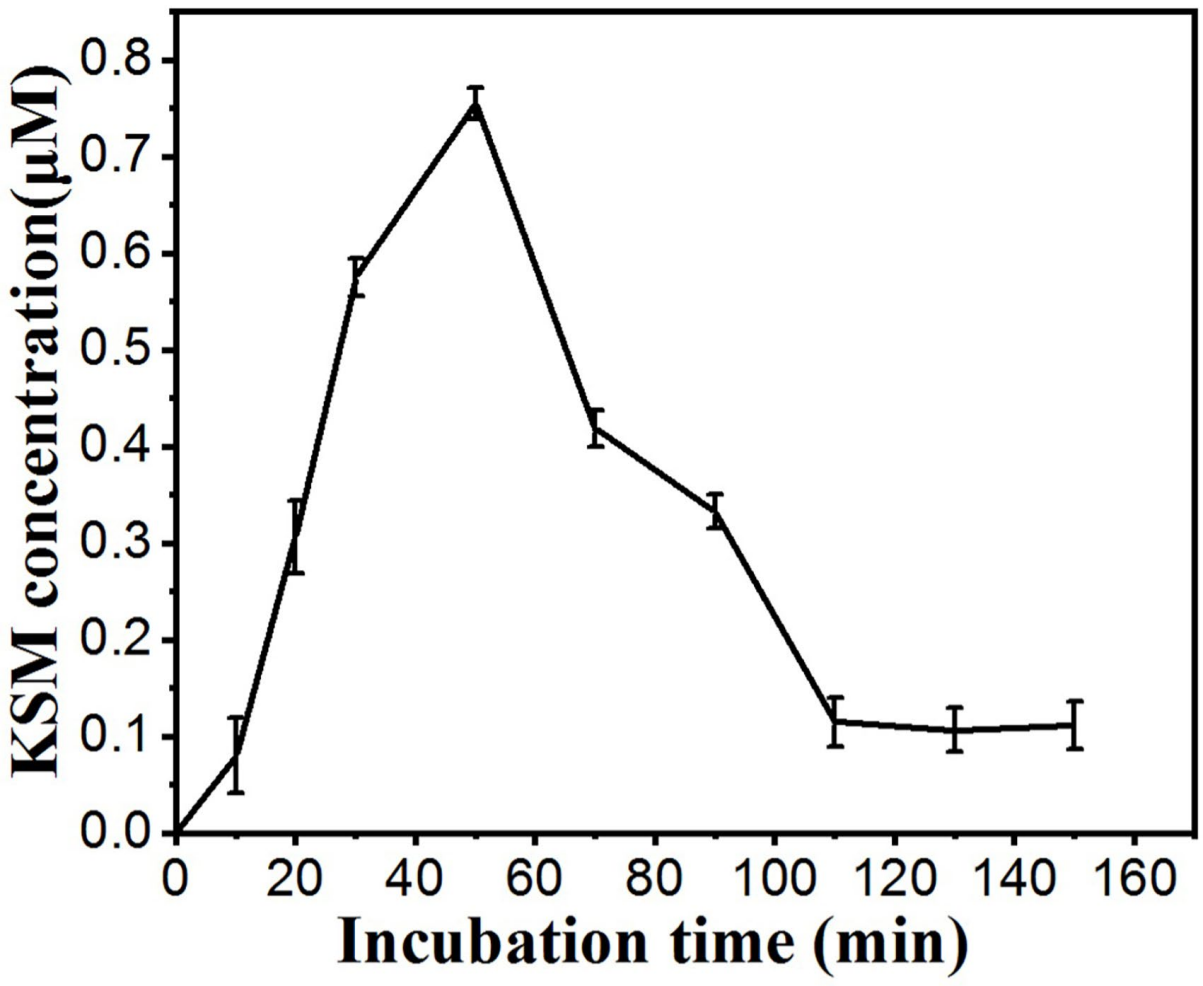
Time course of the KSM concentration in the phloem exudate of castor bean. The cotyledons were incubated in a buffered solution containing 0.1 mM KSM (pH 5.5). The hook was severed at time 1 h, and then the exudate was collected from 10 to 150 min (mean of three sets of 8 plants ± SE).

Under our experimental conditions, KSM accumulated in the phloem exudate. Time-course experiments indicated that the KSM concentration in the phloem exudate increased for 60 min and then decreased (Figure 5). The concentration of KSM in the exudate from 0 to 50 min increased linearly over time, while the content of KSM over 60 to 90 min showed a downward trend. The reason for this may be that the vitality of the plants gradually diminished under *in vitro* conditions, while the concentration of the KSM solution decreased continuously.

### Concentration dependence uptake of KSM by castor bean cotyledons

**Figure 6:**
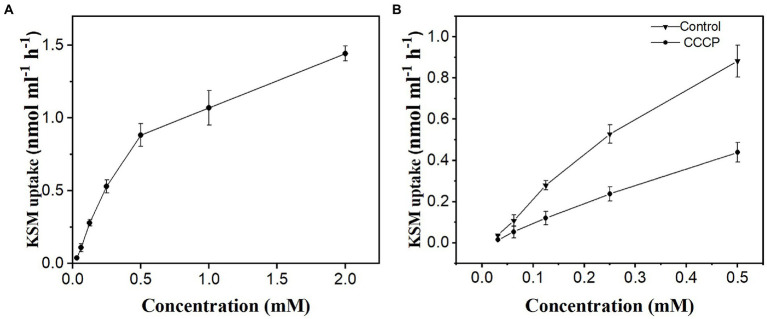
Concentration dependence of KSM in phloem exudate of castor bean seedlings. Phloem exudate was collected for 1 h after cotyledons were preincubated for 1 h in KSM medium **(A)** concentration range from 0.01625 to 2 mM; **(B)** complementary set, without (control) or with 50 μM CCCP. Each point is the mean of three sets of 8 seedlings ± SE.

To investigate the concentration dependence of KSM uptake, the phloem exudate of castor bean was collected after cotyledon incubation with KSM at concentrations ranging from 0.01625 to 2 mM. The results showed that there were two components involved in KSM uptake, a saturable component at lower concentrations (from 0.01625 to 0.5 mM) and a nonsaturable component at higher concentrations (Figure 6A). The saturable component observed at the lowest concentrations was abolished by 50 mM CCCP. Active uptake represents 49%, 40%, and 42% of the total influx at concentrations of 0.0625, 0.125, and 0.25 mM, respectively (Figure 6B).

**Figure 7:**
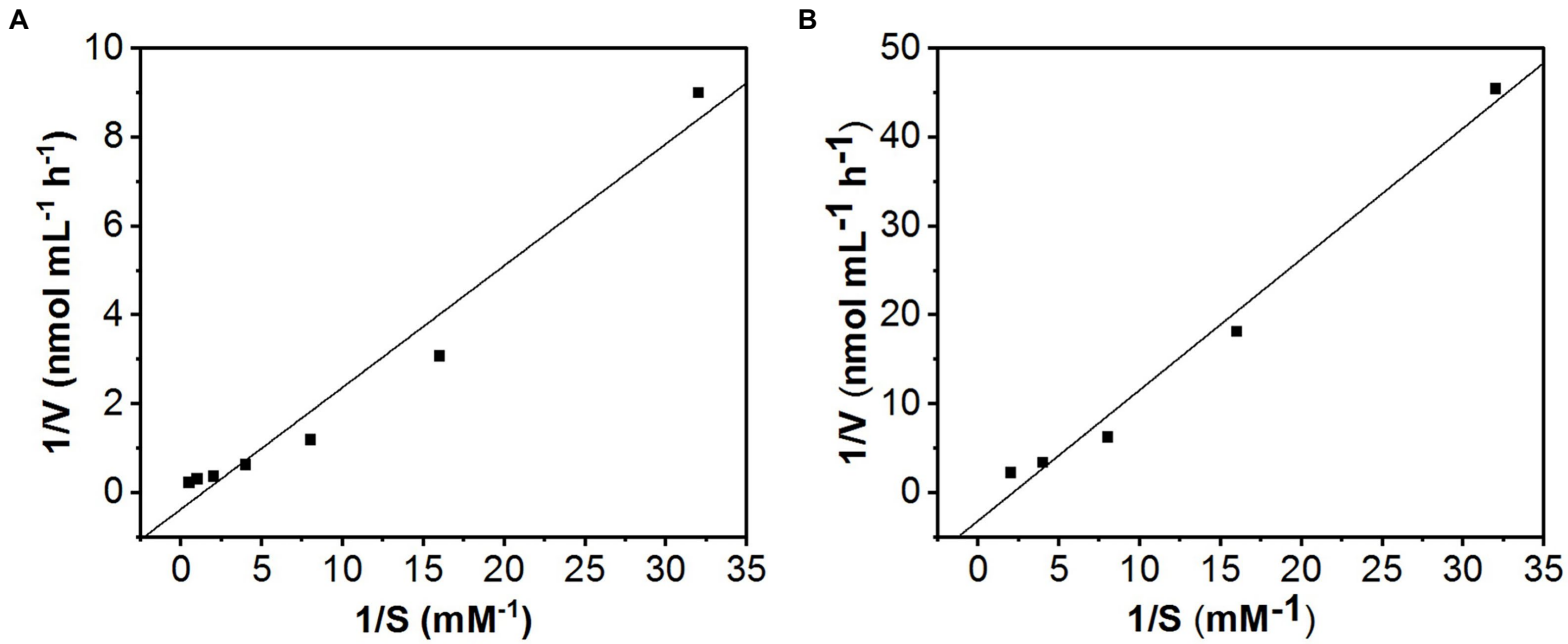
Lineweaver−Burk plots of KSM uptake. The kinetic data were calculated from the slope and the intercept of the Lineweaver−Burk plot. **(A)** Data from Figure 6A; the line yields a *Km* = 2.7 mM and *Vmax* = 0.75 nmol mL^−1^ h^−1^. **(B)** Data from Figure 5B after subtracting the CCCP-insensitive component; the line yields a *Km* = 0.43 mM and *Vmax* = 0.32 nmol mL^−1^ h^−1^.

The apparent kinetic parameters for total uptake using Lineweaver–Burk plots were *Km* = 2.7 mM and *Vmax* = 0.75 nmol mL^−1^ h^−1^ (Figure 7A). After subtracting the passive component from the total amount of KSM uptake, the kinetic parameters were *Km* = 0.43 mM and *Vmax* = 0.32 nmol mL^−1^ h^−1^ (Figure 7B).

### Effect of pH and temperature on the uptake of KSM by castor bean cotyledons

**Figure 8:**
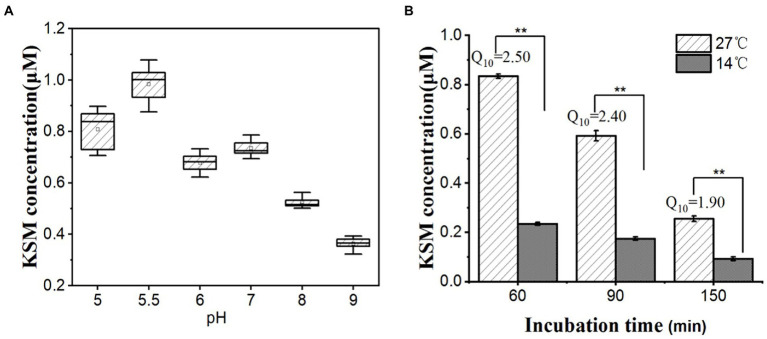
Effect of pH and temperature on 0.1 mM KSM uptake by cotyledons from the castor bean. **(A)** Cotyledons were incubated in 0.1 mM KSM for 1 h (pH range from 5 to 9) for 60 min. **(B)** Cotyledons were incubated in 0.1 mM KSM (pH 5.5) and then placed at 14°C or 27°C for 60, 90, and 150 min. The figure indicates that the data (mean of three sets of 8 plants ± SE) within a column are significantly different by Duncan’s multiple range test (^**^p < 0.01).

The uptake of xenobiotics by plants is affected by many factors, such as physical and chemical properties xenobiotics and the characteristics of the crop. To study the pH effect on KSM uptake, the cotyledons were soaked in buffer solutions with different pH values (pH ranging from 5.0 to 9.0). The uptake of KSM was sensitive to pH (Figure 8A). Different pH values can affect the dissociation of the compound, thereby affecting the uptake. At pH = 5.5, the uptake was 1.03 μM, and with increasing pH, the uptake of KSM by castor bean gradually decreased. At pH values of 6, 7, and 8, the uptake was 0.72, 0.77, and 0.55 μM, respectively. At pH = 9.0, the uptake was significantly inhibited and was only 36.9% of that at pH = 5.5. The above results confirmed that KSM uptake at lower concentrations is proton motive force-dependent. Conceivably, differences in pH could induce physicochemical changes in the tissue, which might influence its permeability to KSM, but the strong dependence on pH speaks against purely passive entry.

The temperature coefficient (Q_10_) represents the factor by which the rate of a reaction increases for every 10° rise in the temperature. For many biological processes, particularly those that involve large-scale protein conformational changes, Q_10_ values are generally between 2 and 3. Thus, Q_10_ values are used to infer mechanistic insight into the physiological process under investigation (Ruby et al., 1999). To further understand the uptake mechanism, the effect of temperature on KSM uptake was investigated. The uptake of KSM at a low temperature (14°C) was decreased significantly compared with normal incubation conditions (27°C) within 60–90 min, and the Q_10_ was between 2 and 3 (Figure 8B).

### Effect of energy inhibitors on uptake of KSM dinitrophenol

**Figure 9:**
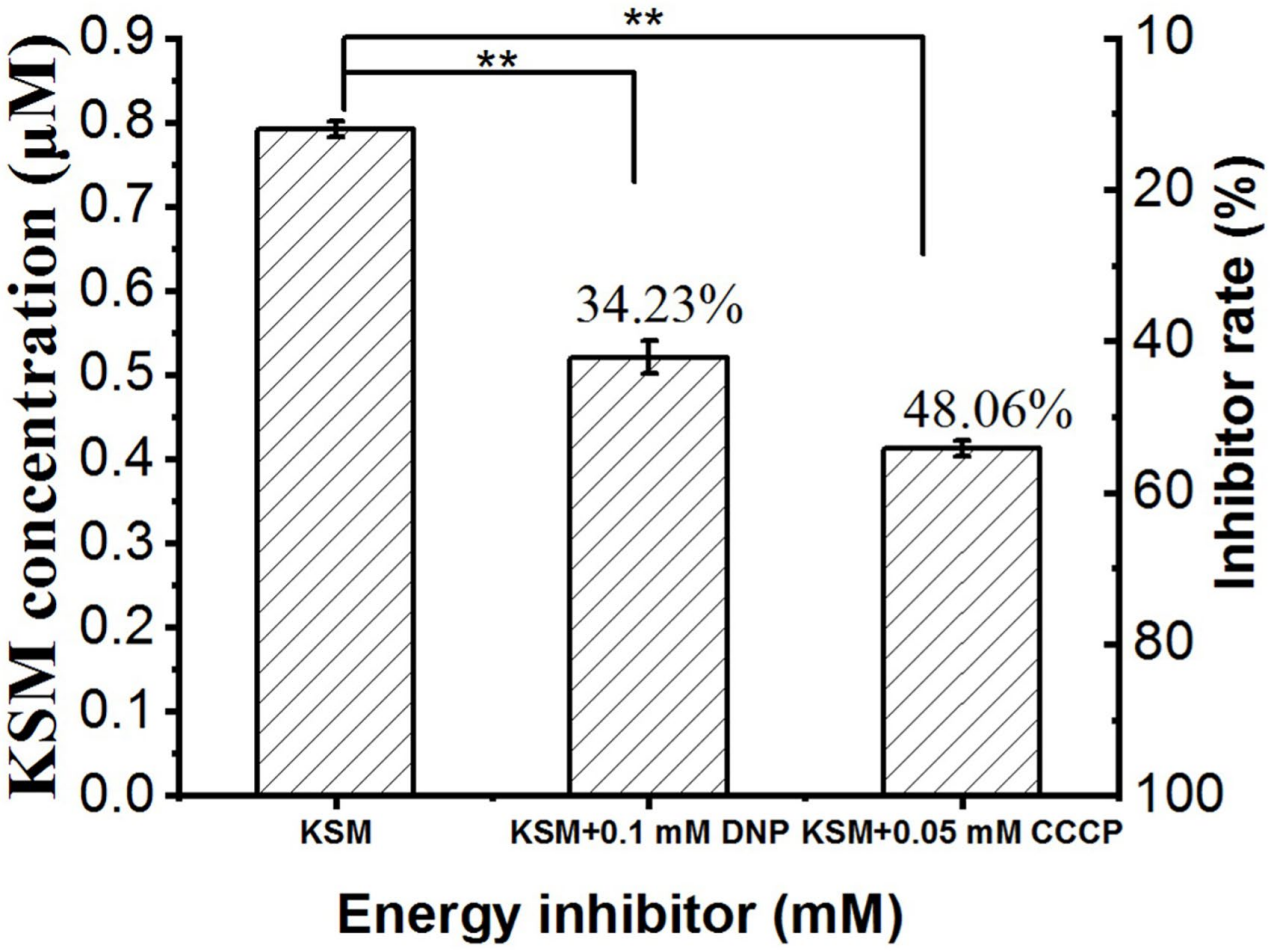
Effect of DNP and CCCP on 0.1 mM KSM uptake by castor bean. Cotyledons were preincubated in a buffered solution at pH 5.5 for 30 min and then transferred to incubation medium containing 0.1 mM KSM for 1 h without (control) or with 0.1 mM DNP or 0.05 mM CCCP. The figure indicates that the data (mean of three sets of 8 plants ± SE) within a column were significantly different by Duncan’s multiple range test (^**^p < 0.01).

Dinitrophenol (DNP) and CCCP can significantly inhibit the uptake of aminoglycoside antibiotics by bacteria, indicating energy-consuming active uptake (Gomes et al., 2005). Therefore, we used the energy inhibitor 0.05 mM CCCP and 0.1 mM DNP to pretreat cotyledons for 1 h and then collected the phloem exudate. The results showed that after treatment with 0.05 mM CCCP and 0.1 mM DNP, the concentration in the phloem exudate was remarkably decreased to 34.23% and 48.06% of the control (0.78 μM), respectively (Figure 9). This result indicated that the uptake of KSM was an energy-dependent process.

### Effect of competitive substrates on the uptake of KSM

**Figure 10:**
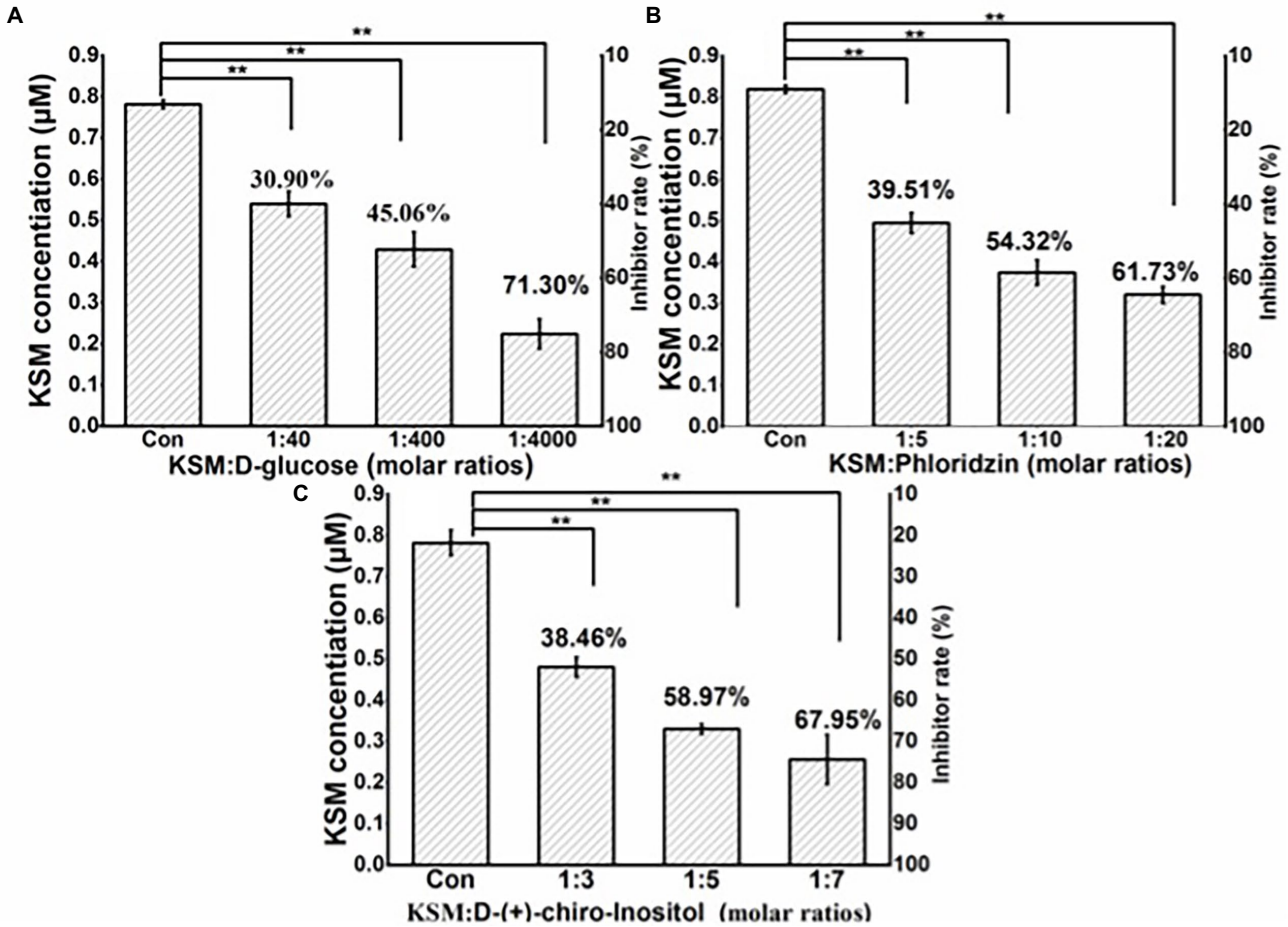
Effect of D-glucose **(A)**, phloridzin **(B)**, and D-chiro-inositol **(C)** on 0.1 mM KSM uptake by castor bean. Cotyledons were preincubated in a buffered solution at pH 5.5 for 30 min and then transferred to incubation medium containing 0.1 mM KSM for 1 h without (control) or with different molar ratios of glucose, phloridzin and D-chiro-inositol. The figure indicates that the data (mean of three sets of 8 plants ± SE) within a column were significantly different by Duncan’s multiple range test (^**^p < 0.01).

Glucose inhibited the uptake of KSM, but it was not obvious, the inhibition rates were 30.90%, 45.06%, and 61.73% at molar ratios (KSM: glucose) of 1:40, 1:400, and 1:400, respectively (Figure 10A). Phloridzin showed a moderate inhibitory effect, and the inhibition rates were 39.51%, 54.32%, and 61.73% at molar ratios (KSM: phloridzin) of 1:5, 1:10, and 1:20, respectively (Figure 10B). The inhibition effect of DCI was most vigorous, and the inhibition rates were 38.46%, 58.97%, and 67.95% at molar ratios (KSM: DCI) of 1:3, 1:5, and 1:7, respectively (Figure 10C). Previous studies have shown that KPT2 cell uptake of gentamicin, an aminoglycoside antibiotic, was mediated by the sodium-glucose transporter SGLT2 and could be inhibited by phloridzin and D-glucose (Stubbs et al., 2004), which is consistent with the results of this study.

## Discussion

Castor bean is a symplastic-apoplastic loader, meaning that endogenous molecules from the foliage or exogenous molecules from the incubation solution found in the phloem exudate *via* the symplastic pathway may originate from the transfer cells of the lower epidermis. Many factors affect the membrane permeability of compounds, including the compound size, water solubility, ionicity (*pKa*), and lipophilicity (*logP*). The “Kleier model” has been widely employed to predict the mobility of xenobiotics based on their physicochemical properties (*logP* and *pKa*) and this suggests that when pKa is between 3 and 6 and logP is between −0.5 and 4, xenobiotics may have phloem conductivity (Kleier and HSu, 1996; Chollet et al., 2004). Calculated by software ACD/Percepta v2020.1.0, the *logP* and *pKa* of KSM are −2.06 and −0.62, respectively, ranging into the nonphloem-mobile area. However, KSM could be detected in the roots of 14-day-old castor bean seedlings. This result is inconsistent with the prediction model, but the target compound can be detected in the root, indicating a carrier-mediated uptake mechanism. Based on this, a study on the uptake mechanism was carried out in the following step. This indicates that the epidermal cells of the cotyledons can uptake KSM from the culture medium, reach the mesophyll cells of the apical leaf after being transported through the apoplast loading process, and can also reach the vascular bundles of the stem after being transported by the symplastic loading process and parenchyma cells, secondary xylem and ring phloem of the root (Rocher et al., 2006). This shows that KSM uses a mixed method of transport, upward through the xylem and downward through the phloem in castor bean.

An extracellular acidic environment can promote the ability of bacteria and mammalian cells to absorb aminoglycosides (Yoshioka et al., 1996). In the present experiment, the uptake of KSM by castor bean was significantly different under different pH treatments, and the content was the highest at pH = 5.5. Most carrier transport is energy-dependent and depends on the proton dynamic potential established by the plasma membrane H^+^-ATPase (Etxeberria et al., 2005). The external pH causes the proton concentration to change, and the proton concentration change affects the uptake of xenobiotics. Therefore, the uptake of KSM may be a carrier-mediated active process. It can be concluded that an active process is involved in the uptake of KSM by castor bean. Low temperature reduces the activity of mitochondrial ATPase and pyruvate kinase required for the synthesis of ATP so that the level of adenylate, especially ATP, decreases significantly, leading to disturbances in the metabolic process. Studies have shown that low temperature can inhibit the active uptake of silicon by cucumbers and phenanthrene in wheat and ATP-binding protein carriers or a process driven by the H^+^-ATP pump (Felle et al., 1983; Steyger et al., 2003). An inhibitor of oxidative phosphorylation can inhibit the energy production of cells. Studies have shown that DNP can inhibit the uptake of iodate by plants (Lu et al., 2016). CCCP can affect the uptake of compounds by plants by removing the proton driving force in the transmembrane process and it is an uncoupling agent, which is an important criterion for judging whether the uptake of xenobiotics requires energy and has been widely utilized in the study of transmembrane transport (Xu et al., 2019). Jiang et al. (2018) used CCCP to prove that the uptake of glycine–fipronil conjugate by castor bean is an energy-consuming process. Similar to previous studies, both CCCP and DNP in this study had inhibitory effects on the absorption of KSM in castor beans, indicating that the absorption of KSM requires energy consumption.

Based on the above results, we can confirm that the uptake of KSM is mediated by a certain kind of carrier. There is a D-chiro-inositol (DCI) moiety in the chemical structure of KSM. DCI belongs to a group of monosaccharides in plants. Developing garden pea embryos were able to take up exogenously applied DCI, and the competition for the uptake of DCI by glucose and sucrose and the susceptibility to CCCP suggested that a carrier was involved in its uptake (Lahuta and Dzik, 2011). Phloridzin is an inhibitor of glucose transporters and it is also used as an inhibitor of hexose transporters and DCI (Ehrenkranz et al., 2005). Competitive substrates of glucose, phloridzin, and DCI were used in this study to preliminarily explore the carrier that mediated uptake of KSM by castor bean cotyledons. Based on the strong effect of DCI and phloridzin, we preliminarily concluded that one or some inositol transporters mediate the uptake of KSM. Inositol transporters belong to the sugar transporter superfamily. Sugar transporters in plants are a very large family, and it is complicated to explore the relationship between sugar transporters and substrates. The first plant inositol transporters were identified in *Mesembryanthemum crystallinum,* but the mechanism was not analyzed in detail (Chauhan et al., 2000). The best characterized transporters to date are those from *Arabidopsis thaliana*. AtINT2 and AtINT4 were both shown to be H^+^/inositol symporters with myo-inositol, scyllo-inositol, and DCI transport functions. Many additional genes coding for putative inositol transporters have been identified in a variety of plant species, but the transport characteristics as well as the physiological roles of the encoded proteins are waiting to be discovered (Schneider, 2015).

Based on homology with *Arabidopsis thaliana* sugar transporters, the castor bean genome was predicted to harbor 53 genes encoding sugar transporters, falling into the eight previously defined subfamilies INT, PMT, VGT, STP, ERD6, pGlucT, TMT, and SUT. INT is responsible for inositol transport, and there are six INTs in castor beans, but their mechanism is still not clear (Mao et al., 2017). We will identify specific transporters using transcriptome, prokaryotic expression, and hyperexpression techniques in future studies. Carrier-mediated pro-pesticides are an innovative strategy for the development of new pesticides in the future. After conjugating a substrate (α-amino acids or sugars) of plant transporters with a nonsystemic pesticide, the resulting conjugate may be actively transported across the plasma membrane by transporters. With this strategy, the conjugates are expected to be delivered to the target organs of the substrate. Many studies have been carried out based on this strategy, especially in the design of phloem-mobile pesticides. Phloem-mobile pro-nematicide, a hydroxymethyloxamyl glucuronide, exhibited root-specific activation in transgenic tobacco (Hsu et al., 1995). The uptake of 2,4 D-Lys by broad bean leaf discs is mediated by an active carrier system (Delétage-Grandon et al., 2001). *Ricinus communis* monosaccharide transporters, *RcSTP1*, mediate the uptake of glucose–fipronil conjugate (Mao et al., 2017). Uptake of glutamine–fipronil conjugate is mediated by *Arabidopsis thaliana* lysine histidine transporter (AtLHT1; Jiang et al., 2018). A series of monosaccharide-fipronil conjugates showed phloem mobility in castor bean (Wu et al., 2019; Wang et al., 2022). Phenazine-1-carboxylic acid-valine conjugate possess good phloem transport in tobacco and promising *in vivo* antifungal activity against *Rhizoctonia solani* Kühn (Zhu et al., 2022).

Carrier-mediated pro-pesticide strategies face many challenges, such as little is known about the carriers and the loss of biological activity and stability during long-distance translocation. Clarifying the relationship between the structure of pesticides and conductivity is conducive to the design and development of targeted pesticides. Therefore, it is essential for developing carrier-mediated pro-pesticide strategies to study the uptake and transport characteristics of KSM in this study.

## Data availability statement

The original contributions presented in the study are included in the article/supplementary material, further inquiries can be directed to the corresponding author.

## Author contributions

AH contributed to the conception of the study. HZ and XX performed the experiment and wrote the manuscript. CZ performed the data analyses. QZ, WZ, and GW helped to perform the experiment. All authors contributed to the article and approved the submitted version.

## Funding

This work was supported by the Natural Science Foundation of China (grant no. 31760531), Chinese Undergraduate Innovation and Entrepreneurship Training Program (grant no. 202110657015; contract no. Guizhou University 2021[016]) and Guizhou High-level Innovative Talents Project (grant no. GCC[2022]027-1).

## Conflict of interest

The authors declare that the research was conducted in the absence of any commercial or financial relationships that could be construed as a potential conflict of interest.

## Publisher’s note

All claims expressed in this article are solely those of the authors and do not necessarily represent those of their affiliated organizations, or those of the publisher, the editors and the reviewers. Any product that may be evaluated in this article, or claim that may be made by its manufacturer, is not guaranteed or endorsed by the publisher.
